# C22 disrupts embryogenesis and extends *C. elegans* lifespan

**DOI:** 10.3389/fphys.2023.1241554

**Published:** 2023-09-18

**Authors:** Safa Beydoun, Aditya Sridhar, Angela M. Tuckowski, Emily Wang, Scott F. Leiser

**Affiliations:** ^1^ Molecular and Integrative Physiology Department, University of Michigan, Ann Arbor, MI, United States; ^2^ Molecular, Cellular and Developmental Biology Department, University of Michigan, Ann Arbor, MI, United States; ^3^ Cellular and Molecular Biology Program, University of Michigan, Ann Arbor, MI, United States; ^4^ Department of Internal Medicine, University of Michigan, Ann Arbor, MI, United States

**Keywords:** *C. elegans*, lifespan, embryogenesis, FUdR, C22

## Abstract

*Caenorhabditis elegans* is an instrumental model in aging research due to its large brood size, short lifespan, and malleable genetics. However, maintaining a synchronous nematode population for longevity studies is challenging and time consuming due to their quick rate of development and reproduction. Multiple methods are employed in the field, ranging from worm strains with temperature dependent sterility to DNA replication inhibitors such as 5′-fluorodeoxyuridine (FUdR). In this study, we characterize a small molecule (C22) that impairs eggshell integrity and disrupts early embryogenesis to determine its applicability as a potential FUdR alternative. We find that C22 prevents egg hatching in a concentration dependent manner. However, it extends the lifespan of wild type worms and can induce FMO-2, a longevity regulating enzyme downstream of dietary restriction. Our results suggest that C22 is unlikely to be widely useful as an alternative to FUdR but its mechanism for lifespan extension may be worth further investigation.

## Introduction

Aging is the major risk factor for multiple chronic diseases, including diabetes, cancer, and Alzheimer’s disease, making it a prime therapeutic target. Despite its ubiquity, much remains to be understood about the molecular mechanisms that underlie the aging process. As a result, a wide range of model organisms are employed in aging research, including the nematode *Caenorhabditis elegans*.

*C. elegans* exhibits many traits that make it an excellent candidate for longevity studies. The nematode has a well-understood cellular makeup and genome, in addition to having multiple phenotypes associated with aging (Garigan et al., 2002; Olsen et al., 2006; Chew et al., 2017). Several established longevity pathways, including dietary restriction and insulin signaling, extend lifespan in *C. elegans*, demonstrating the translational potential of the organism (Kenyon et al., 1993; Kimura et al., 1997; Greer and Brunet, 2009). Furthermore, the hermaphrodites of the species rapidly produce large numbers of genetically identical offspring, making *C. elegans* maintenance relatively straightforward. However, this poses challenges in longevity studies, as aging experiments in *C. elegans* involve monitoring the survival of a synchronized population to identify interventions that modify lifespan. Since *C. elegans* offspring quickly reach adulthood, they can become indistinguishable from the parental generation, contaminating the experimental cohort.

To mitigate this issue, multiple techniques have been utilized, each with their own sets of caveats. These techniques include but are not limited to: 1) separating adults from larva daily during their reproductive phase, 2) chemical sterilizing agents (Wang et al., 2019), 3) RNAi inducing embryonic arrest (Burton et al., 2011), and 4) worm strains that have temperature-dependent fertility/sterility (Johnston et al., 2008). Moving adults daily until their reproduction ceases is a highly implemented technique. However, this technique is time-consuming and technically challenging, in addition to increased handling of the worm and the important caveat in dietary restriction (DR) studies, where starvation induces facultative vivipary (Chen and Caswell-Chen, 2004), also known as internal hatching. To induce sterility and prevent internal hatching, the sterilizing agent 5′-fluorodeoxyuridine (FUdR) (Wang et al., 2019) can be added to the nematode growth media (NGM). However, exposure of larval worms to FUdR results in stunted growth and reduced lifespan, while exposure at adulthood leads to increased lifespan, thus implicating FUdR itself in longevity regulation (Wang et al., 2019). *pos-1* RNAi is another method used to induce embryonic arrest (Burton et al., 2011). POS-1 is a CCCH zinc-finger RNA-binding protein that regulates cell-fate specification during embryogenesis in *C. elegans* (Tabara et al., 1999; Elewa et al., 2015). Exposure of test populations (usually from egg) to *pos-1* RNAi before the onset of embryogenesis results in embryonic arrest of the progeny. However, this technique limits the use of other RNAi, as the combination of multiple RNAi can result in varying knockdown effects and variable results. Lastly, strains with temperature dependent fertility/sterility (Johnston et al., 2008) have also been employed in the field where fertility is maintained at 15°C and blocked at 25°C. A mutation in the *fem-1* gene [*fem-1* (*hc17*)] blocks spermatogenesis at 25°C (Spence et al., 1990) while mutations in the *glp-1*[*glp-1* (*e2141*)] (Kodoyianni et al., 1992) or *glp-4* [*glp-4* (*bn2*)] (Beanan and Strome, 1992) genes block germline proliferation when shifted to 25°C. The main issue in using these strains is the effect of temperature changes on worm lifespan and physiology (Miller et al., 2017; Vakkayil and Hoppe, 2022). In this study, we characterize a novel compound, C22, that was identified from a screen of small molecules that disrupt embryogenesis (Weicksel et al., 2016), as a potential alternative to induce sterility in longevity studies in *C. elegans.* We find that while C22 is effective in disrupting embryogenesis, it also alters various measures of health and longevity. Thus, C22 is an unlikely alternative for FUdR, but has intriguing properties worth exploring.

## Materials and methods

### Strains and growth conditions

Standard *C. elegans* cultivation procedures were used as previously described (Kaeberlein et al., 2006; Smith et al., 2008). Briefly, N2 wild type, VC1668 [*fmo-2*(ok2147)], and LZR1 (Miller et al., 2022) (allele hamSi1) [(pCF150) (*fmo-2*p::mCherry + H2B::GFP) + Cbr-unc-119(+)] II strains were maintained on solid nematode growth media (NGM) using *E. coli* OP50 throughout life and housed in a 20°C Percival incubator. All experiments were conducted at 20°C unless stated otherwise.

### Interventions

Nematode growth media (NGM) was autoclaved and then cooled to 55°C prior to the addition of various amounts of C22 (Hit2Lead #9345554) or equal volumes of DMSO controls or DMSO/FUdR. Stocks of 12.5 mM C22 dissolved in DMSO and 150 mM FUdR dissolved in water were used. The final concentration of DMSO in plates varied based on the concentrations of C22 tested. When making 3, 5, 15, 25, 50, and 100 µM C22 plates, corresponding 0.024%, 0.04%, 0.12%, 0.2%, 0.4%, and 0.8% DMSO were added to control plates. The final concentration of FUdR was 50 μM. Fresh batches of plates were poured 1 week before the onset of each experiment and stored at 4°C protected from light. Plates were seeded with select bacteria 2–3 days before the onset of experiments.

### Food source

Animals were fed live or dead (Beydoun et al., 2021) *E. coli* OP50. Live food: A single colony of bacteria was inoculated in 500 mL Luria broth (LB) and cultured overnight (∼14 h) in a 37°C shaker incubator. Stock plates were seeded with 200 μl bacteria (OD_600_ 3.0). For lifespan plates containing carbenicillin, the bacteria were transferred to 50 mL conical tubes and centrifuged at 3,000 g for 20 min. The plates were then seeded with 200 μl bacteria (OD_600_ 3.0) concentrated 5x. The protocol for dead bacteria was as previously described (Beydoun et al., 2021). Briefly, a single colony of bacteria was inoculated in 500 mL Luria broth (LB) and cultured overnight (∼14 h) in a 37°C shaker incubator. 32% paraformaldehyde (PFA) was then added to the cultured bacteria (OD_600_ 3.0) to bring the final concentration to 0.5%. The flask was placed in the 37°C shaker incubator for 1 h to kill the bacteria. The dead bacteria were transferred to 50 mL conical tubes where they were washed 5 times to remove any residual PFA. The plates were then seeded with 200 μl bacteria (OD_600_ 3.0) concentrated 5x in LB.

sDR plates: A single colony of bacteria was inoculated in 500 mL Luria broth (LB) and cultured overnight (∼14 h) in a 37°C shaker incubator. The bacteria were transferred to 50 mL conical tubes and centrifuged at 3,000 g for 20 min. The supernatant was discarded, and the pellet was resuspended in S-media (Stiernagle, 2007) (1L S-basal, 10 ml 1M potassium citrate pH 6.0, 10 ml trace metals solution, 3 ml 1M CaCl_2_, 3 ml 1M MgSO_4_. Add components using sterile technique and filter sterilize.). The fed control was concentrated 5x from an OD_600_ 3.0 and the sDR group was diluted 0.5x from an OD_600_ 3.0. The plates were seeded with 200 μl of respective bacteria.

### Egg hatching

Ten gravid adults were placed on seeded condition (NGM, DMSO, or C22) plates to lay eggs for 1 h at 15, 20, or 25°C. The adults were removed, and the eggs were left to grow until they reached adulthood (F1) and began to lay their own eggs. The effectiveness of the drug was determined by the lack of F2 eggs hatching at this stage. Images that show the F1 adults on a lawn of food surrounded by unhatched F2 eggs indicate that C22 was effective at preventing egg hatching of the progeny. Images that show F1 bagging and F2 hatching indicate that embryogenesis was not disrupted and the F2 progeny was able to develop and grow properly.

### Internal hatching

Fifty day 1 adult hermaphrodites were placed on condition plates (NGM, FUdR, FUdR/DMSO, and C22) and were fasted overnight at 20°C. The number of worms displaying internal hatching was noted the following day and the percentage of worms with internal hatching was determined.

### Lifespans

Synchronization and preparation of animals for lifespan experiments followed previously published techniques (Sutphin and Kaeberlein, 2009). Briefly, 15 gravid adults were placed on new NGM plates or condition test plates. After 4 h the gravid adults were removed and the plates with synchronized eggs were placed back in the 20°C incubator until they reached late L4/young adult (∼2.5 days). Fed conditions- Approximately 60 worms were transferred to fresh plates on days 3, 4, 7, and 10 from egg. sDR conditions- Approximately 60 worms were transferred to fresh fed plates on days 3 and 4 from egg, then transferred to plates with diluted food on days 5, 7, 10, and 12 from egg where they remained for the duration of the lifespan. A minimum of two plates per strain per condition were used per replicate experiment. Experimental animals were scored every 2–3 days and considered dead when they did not move in response to prodding under a dissection microscope. Worms that crawled off the plate were not considered.

### Microscopy

*fmo-2*p::mCherry reporter worms were synchronized by a timed-egg-lay on NGM plates. The animals (*n* = 50) were allowed to develop and were transferred to test plates on day 1 adults and imaged after 24 h. Microscope slides were prepared 1 h prior to microscopy with a 3% agar mount. The worms were immobilized in 10 μL of 30 mM sodium azide placed on the agar pad for 2 min. Pictures were taken immediately after slide preparation using a Leica M165FC dissecting microscope. Fluorescence mean comparisons were quantified in ImageJ (Schneider et al., 2012) bundled with 64-bit Java 1.8.0 using polygon tool and saved as macros. Experimental conditions were normalized to their respective fed DMSO or C22 controls. Data were plotted by R version 4.1.0, Microsoft Excel 365, and GraphPad Prism.

### Pumping rate

Animals were synchronized by placing 10 N2 gravid adult worms on NGM plates seeded with *E. coli* OP50 and allowing them to lay eggs for 2 h at 20°C. The gravid adult worms were then removed, and the eggs were allowed to hatch and grow at 20°C until they reached day 1 adulthood. Worms were transferred to condition plates and the pumping rate was determined after 24 h. The number of contractions of the pharyngeal bulb of 15–20 worms per strain was counted over 30 s. A Leica M205C microscope was used with focus on the pharynx.

### Statistics and reproducibility

One-way ANOVA with Tukey Post-Hoc analysis was used to derive *p*-values for internal hatching and *fmo-2* induction comparisons. A two-tailed *t*-test or One-way ANOVA with Tukey Post-Hoc analysis were used to derive *p*-values for pumping comparisons. Log-rank test was used to derive *p*-value for lifespan comparisons (Han et al., 2016). All error bars shown in the figures represent the standard error of the mean (SEM).

## Results

### C22 inhibits embryogenesis and extends N2 lifespan

**FIGURE 1:**
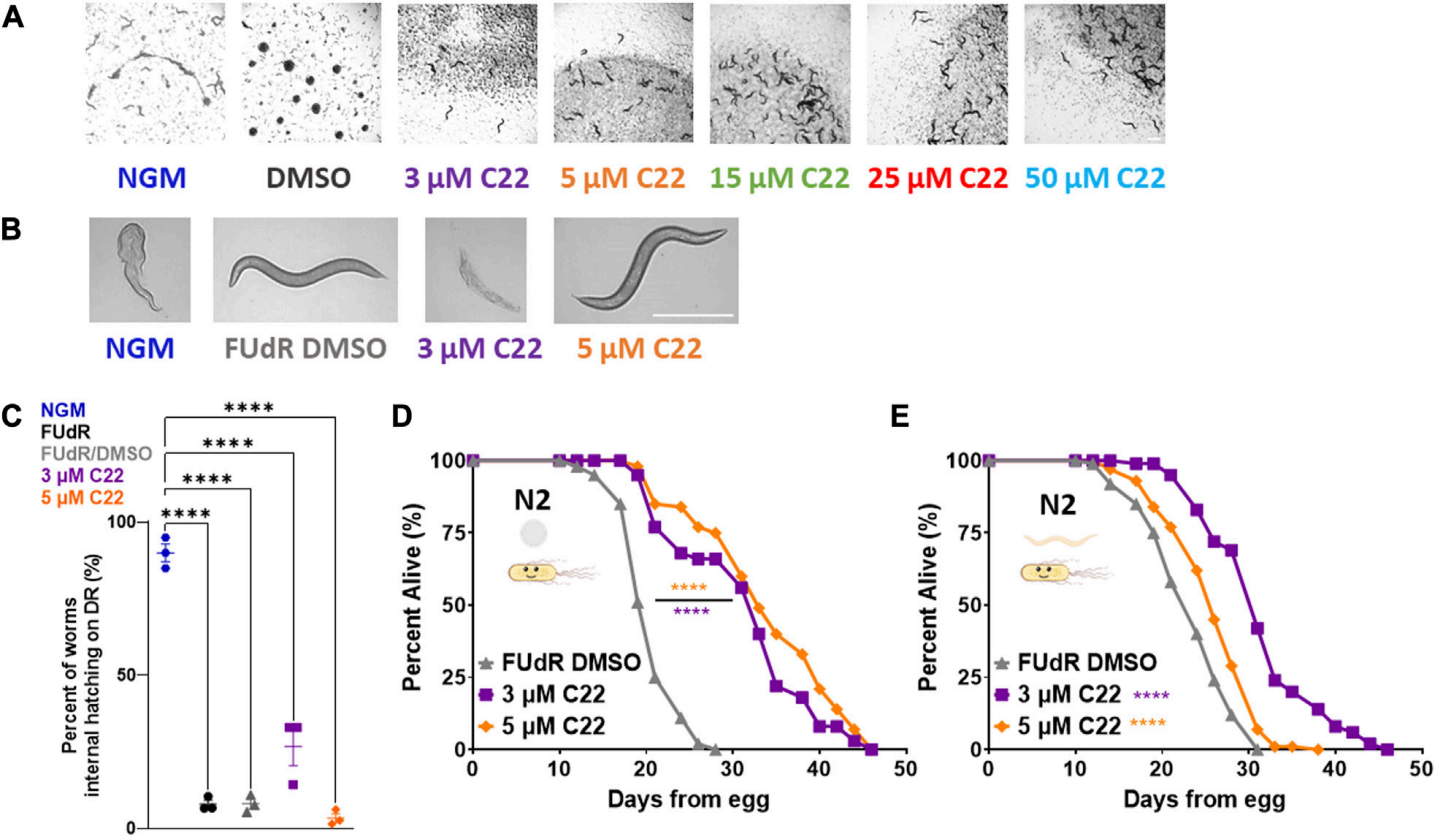
C22 disrupts embryogenesis and extends wildtype *C. elegans* lifespan. **(A)** Images of F2 eggs hatching from F1 worms grown on NGM and DMSO condition plates from egg compared to F2 eggs not hatching from F1 worms grown on C22 test plates from egg at 20°C. Scale bar, 1.0 mm. **(B)** Images of day 1 adult N2 worms on DR displaying internal hatching on NGM and 3 µM C22 but not on FUdR/DMSO and 5 µM C22. Scale bar, 0.5 mm. **(C)** Quantification of percent worms with internal hatching under DR. *N* = 3 experiments. *n* > 30 worms per condition. **(D,E)** Percent alive of N2 worms fed live OP50 on C22 condition plates from **(D)** egg and **(E)** young adulthood. *N* = 2 experiments. *n* ∼ 100 worms per condition. Datasets are available in the source data file. One-way ANOVA with Tukey *post hoc* analysis was used to derive *p*-values for internal hatching comparisons. The log-rank test was used to derive *p*-values for lifespan comparisons. All error bars shown in figures represent the standard error of the mean (SEM) **** denotes *p*-value < 0.0001.

C22 is a small molecule identified to disrupt eggshell formation in *C. elegans* from a screen of thousands of compounds (Weicksel et al., 2016). Prevention of egg-hatching without altering lifespan is highly useful for labs with an interest in longevity regulation in *C. elegans.* To test whether C22 is a suitable candidate for lifespan studies, we first asked whether and at what concentration C22 could prevent eggs from hatching in a lifespan. A previous report (Weicksel et al., 2016) showed that C22, in combination with FUdR, could successfully prevent eggs from hatching. Based on data from that study, we tested various concentrations of C22 for their efficacy in preventing eggs from hatching at 15°C, 20°C, and 25°C. Our results show that C22 disrupts embryogenesis at the tested concentrations and temperatures (Figure 1A; Supplementary Figures S1A, B). Since we did not observe a difference in the effectiveness of C22 at the various temperatures tested, the rest of the assays were conducted at 20°C.

One of the most studied longevity interventions across taxa is dietary restriction (DR). Since starvation induces facultative vivipary (Chen and Caswell-Chen, 2004) we tested whether C22 can be used as an alternative to FUdR to prevent internal hatching under DR. We find that C22 prevents internal hatching under DR in a concentration dependent manner (Figures 1B, C). Preventing egg-hatching under fed conditions (Figure 1A) and facultative vivipary under DR (Figures 1B, C) support C22 as an effective alternative to FUdR in its ability to block egg development in lifespan experiments.

Since many interventions that impact fertility also alter lifespan (Friedman and Johnson, 1988; Mendenhall et al., 2009), we next wanted to determine whether C22 concentrations that inhibit egg hatching also alter lifespan in *C. elegans*. To determine whether C22 affects longevity, we tested the effect of C22 on wild type/N2 lifespan. Interestingly, we find that C22 significantly extends N2 lifespan when administered from egg (Figure 1D) and from young adulthood (Figure 1E). This lifespan increase was significant at both low doses of C22 (3 and 5 μM) but was greater in magnitude when treatment began from egg (Supplementary Table S1). These results suggest that C22 is a viable tool for preventing egg-hatching in lifespan studies ± food, but that it extends lifespan in an exposure time-dependent manner. When considering the effect of DMSO on C22 mediated lifespan extension, we find that C22 increases wildtype lifespan as compared to DMSO only control (Supplementary Figure S2).

### C22 interacts with *fmo-2* mediated longevity regulation

**FIGURE 2:**
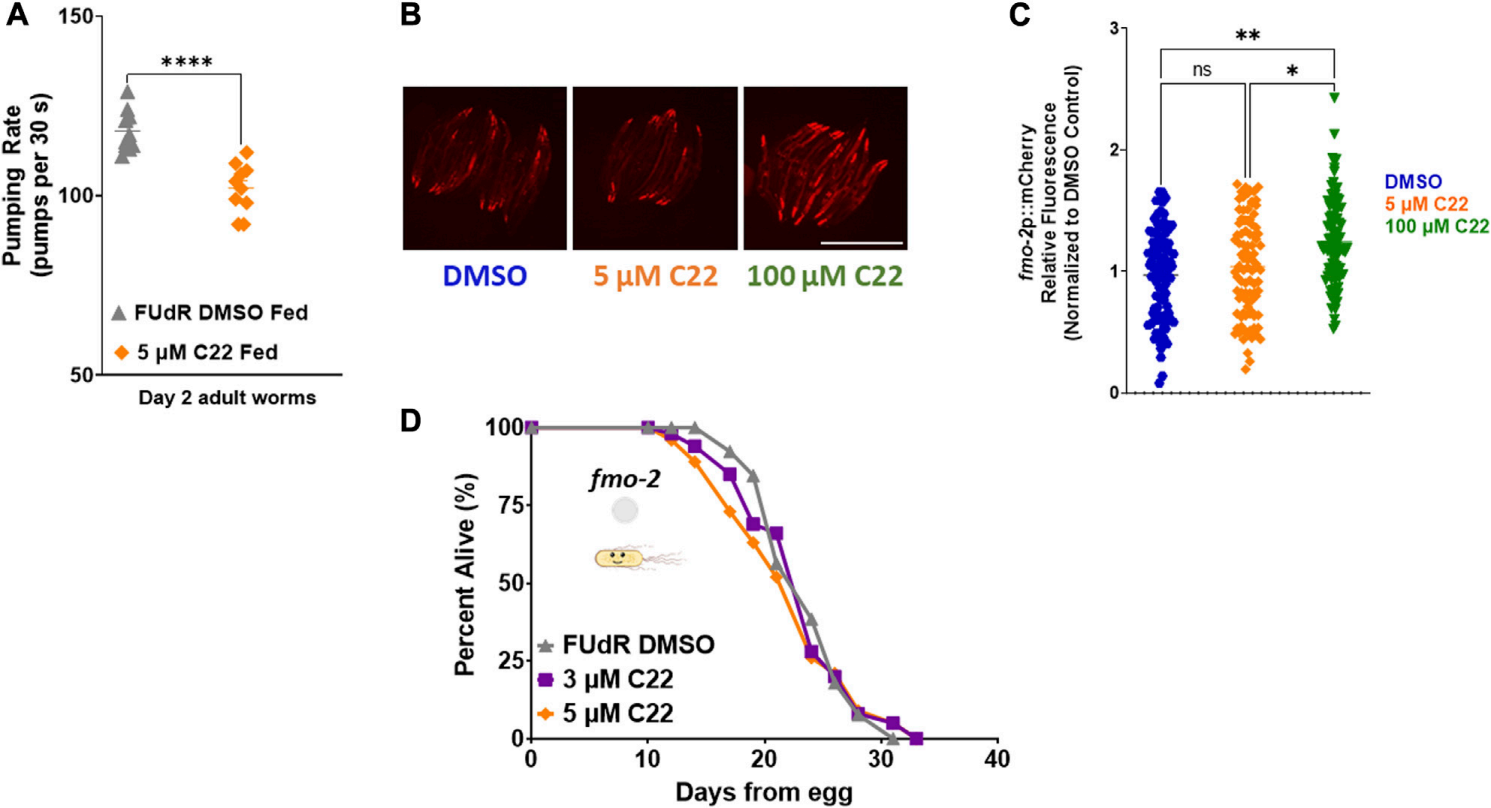
C22 induces *fmo-2* under fed conditions and requires it for lifespan extension. **(A)** Pumping rate (pumps per 30 s) of day 2 adult fed N2 worms on C22 and control conditions. *N* = 2 experiments. *n* = 10 worms per condition. **(B)** Images of *fmo-2* induction in day 2 adult worms after overnight exposure to C22 using the *fmo-2*p::mCherry reporter. *N* = 2 experiments. *n* > 30 worms per condition. Scale bar, 1 mm. **(C)** Quantification of mCherry fluorescence normalized to DMSO control. **(D)** Percent alive of *fmo-2* worms fed live OP50 on C22 condition plates from egg. *N* = 2 experiments. *n* ∼ 100 worms per condition. Datasets are available in the source data file. A two-tailed *t*-test was used to derive *p*-values for pumping rate comparisons. One-way ANOVA with Tukey *post hoc* analysis was used to derive *p*-values for relative fluorescence comparisons. The log-rank test was used to derive *p*-values for lifespan comparisons. All error bars shown in figures represent the standard error of the mean (SEM) ns, no significant difference, * denotes *p*-value < 0.05, ** denotes *p*-value < 0.01, and **** denotes *p*-value < 0.0001.

Having established that C22 alone can prevent egg-hatching and extend lifespan in *C. elegans,* we next wondered whether it acts through previously reported longevity pathways. To ask whether C22 treatment might affect food intake, we measured pumping rate in control and C22 treated animals. We find that there is a small but statistically significant decrease in pumping rate in worms treated with C22 (Figure 2A). Based on this result, we hypothesized that C22 could extend worm lifespan through the dietary restriction pathway. Our previous work identified flavin-containing monooxygenase-2, or *fmo-2,* as a key gene in modulating longevity downstream of dietary restriction (Leiser et al., 2015). Overexpression of FMO-2 is sufficient to increase lifespan in *C. elegans* (Choi et al., 2023; Leiser et al., 2015), and dietary restriction mimetics can induce *fmo-2* and extend *C. elegans* lifespan (Miller et al., 2022) under fed conditions. Since C22 extends fed N2 lifespan (Figures 1D, E) we were interested in its effect on *fmo-2* induction. Using a single copy *fmo-2*p::mCherry reporter (Miller et al., 2022), we find that an acute exposure to a high concentration of C22 induces *fmo-2* (Figures 2B, C) under fed conditions. Since *fmo-2* expression is required for DR-mediated lifespan extension (Leiser et al., 2015), *fmo-2* knockout animals can prevent the longevity benefits of the DR pathway. Similarly, C22 mediated lifespan extension is also fully dependent on *fmo-2* expression (Figure 2D). The results show that loss of *fmo-2* prevents C22 from extending worm lifespan, consistent with this gene playing an important role in C22-mediated longevity.

### C22 and DR function in the same pathway to regulate longevity

**FIGURE 3:**
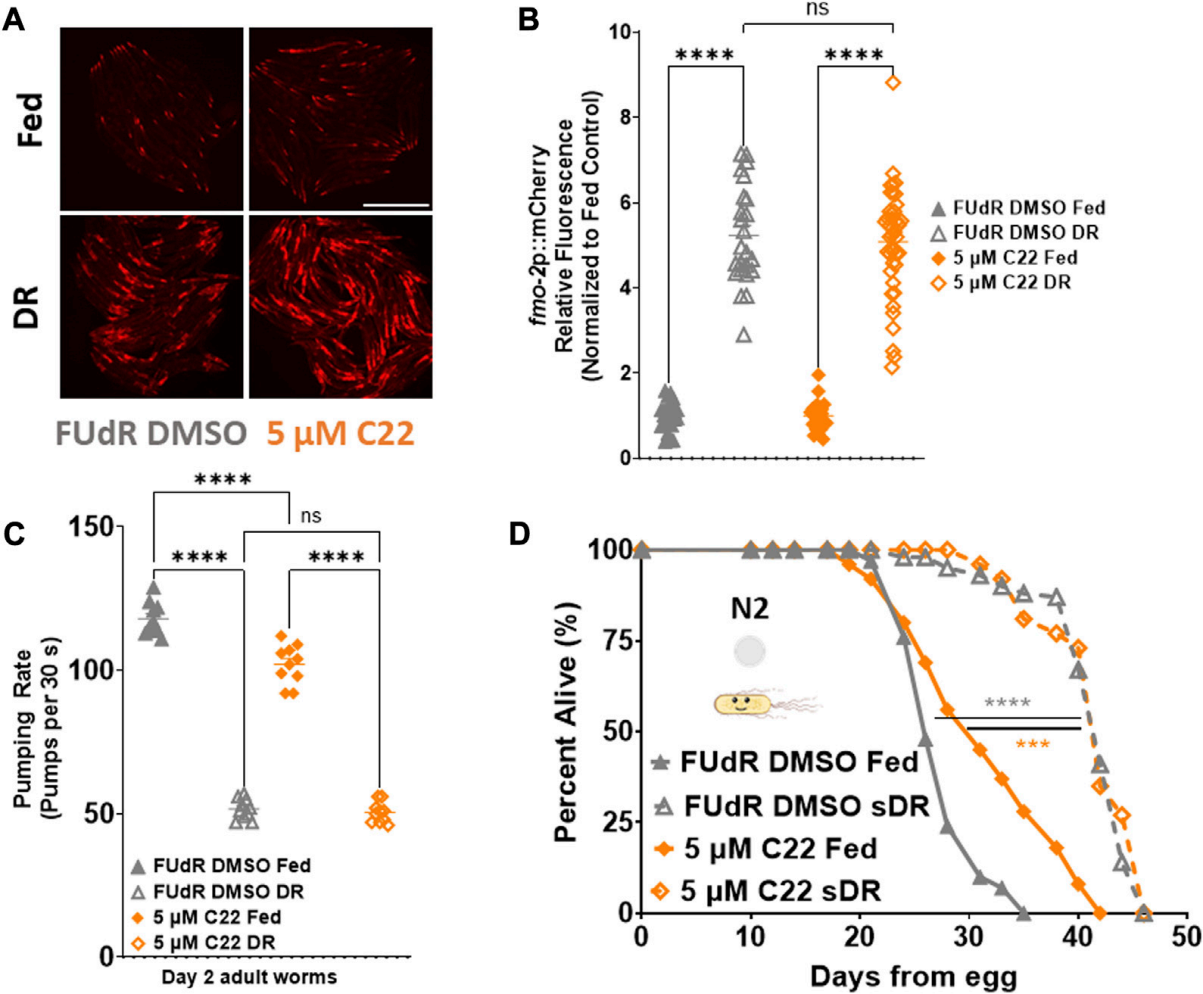
C22 does not further extend DR mediated *fmo-2* induction or lifespan extension. **(A)** Images of *fmo-2* induction in day 2 adult worms after overnight exposure to C22 under fed and DR (overnight fast) using the *fmo-2*p::mCherry reporter. *N* = 2 experiments. *n* > 25 worms per condition. Scale bar, 1 mm. **(B)** Quantification of mCherry fluorescence normalized to fed controls. **(C)** Pumping rate (pumps per 30 s) of day 2 adult DR N2 worms on C22 and control conditions. *N* = 3 experiments. *n* = 10 worms per condition. **(D)** Percent alive of N2 worms on C22 condition plates from egg (separated to fed and sDR groups at Day 3 of adulthood). *N* = 2 experiments. *n* ∼ 100 worms per condition. Datasets are available in the source data file. One-way ANOVA with Tukey *post hoc* analysis was used to derive *p*-values for relative fluorescence and pumping assay. The log-rank test was used to derive *p*-values for lifespan comparisons. All error bars shown in figures represent the standard error of the mean (SEM) ns, no significant difference, *** denotes *p*-value < 0.001, and **** denotes *p*-value < 0.0001.

Seeing as *fmo-2* is necessary downstream of DR and C22-mediated lifespan extension, we hypothesized that C22 would also interact with DR. Dietary restriction is the most well-characterized longevity intervention across taxa (Kaeberlein, 2013). DR is defined as the decreased intake of overall calories and/or specific macronutrients without malnutrition. Since we observed that C22 decreases pumping rate of fed worms (Figure 2A), and that C22 induces *fmo-2* (Figures 2B, C), we were interested in the potential interaction between C22 and DR-mediated longevity. We began by looking at *fmo-2* expression and find that C22 does not further induce *fmo-2* under DR (Figures 3A, B). We next looked at the effect of C22 and DR on feeding. We find that pumping rate is decreased after 24 h on DR and is not further decreased when C22 is combined with DR (Figure 3C). Finally, we looked at the effect of C22 on DR-mediated longevity. We find that C22 and DR increase N2 lifespan, but the lifespan extension of C22 combined with DR is not additive (Figure 3D). These data indicate that C22 and DR likely function in the same pathway to regulate longevity.

### C22 interacts with various measures of *C. elegans* health and longevity partially independent of bacterial metabolism

**FIGURE 4:**
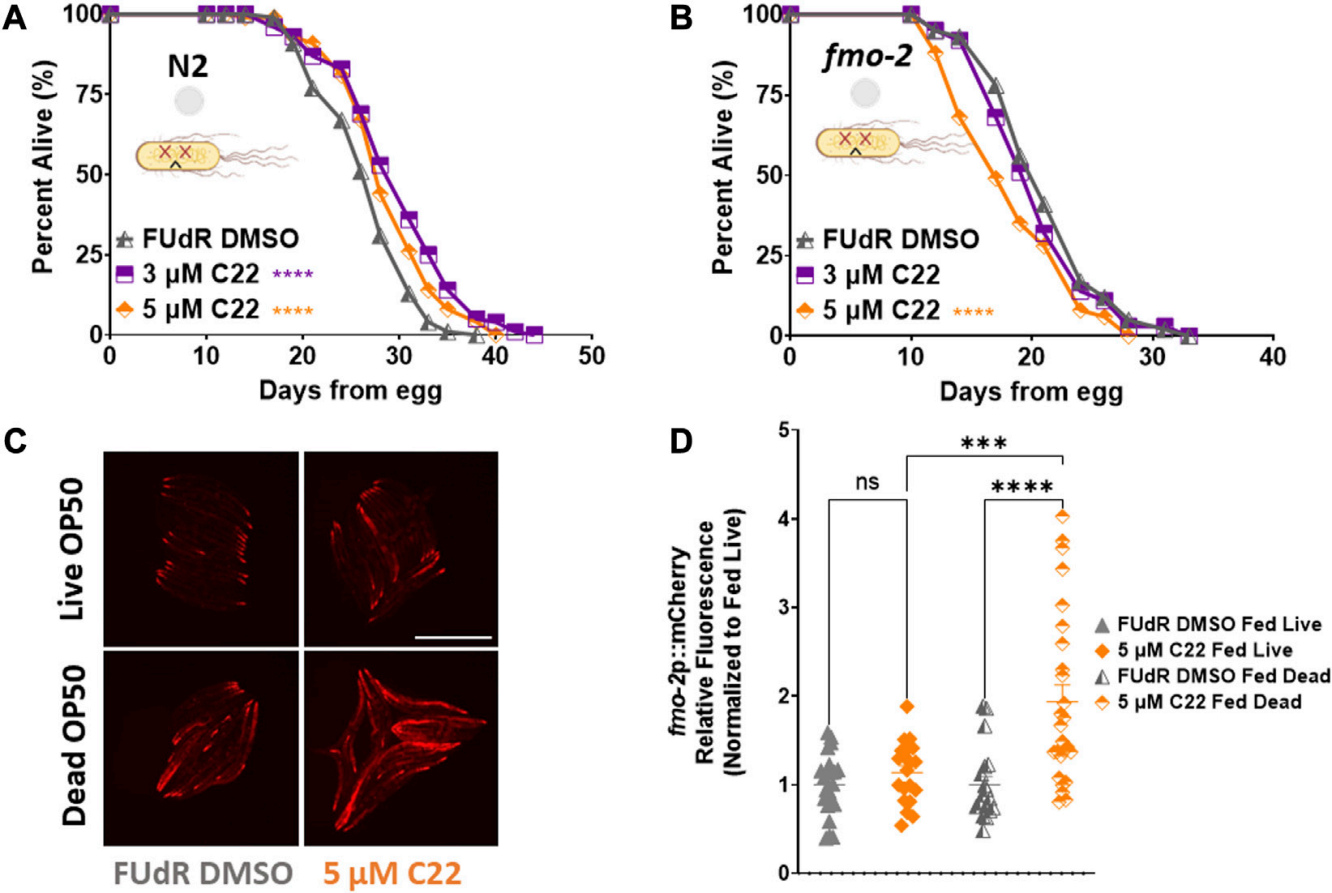
C22 directly impacts the lifespan and healthspan of the worms partially independent of bacteria metabolism. **(A,B)** Percent alive of **(A)** N2 and **(B)** *fmo-2* worms fed dead OP50 on C22 condition plates from egg. *N* = 2 experiments. *n* ∼ 100 worms per condition. **(C)** Images of *fmo-2* induction in day 2 adult worms after overnight exposure to C22 using the *fmo-2*p::mCherry reporter on live and dead OP50. *N* = 2 experiments. *n* > 20 worms per condition. Scale bar, 1 mm. **(D)** Quantification of mCherry fluorescence normalized to fed live controls. Datasets are available in the source data file. The log-rank test was used to derive *p*-values for lifespan comparisons. One-way ANOVA with Tukey *post hoc* analysis was used to derive *p*-values for relative fluorescence comparisons. All error bars shown in figures represent the standard error of the mean (SEM) ns, no significant difference, *** denotes *p*-value < 0.001, and **** denotes *p*-value < 0.0001.

The most common food source for *C. elegans* in the lab is live OP50 *Escherichia Coli*. Since live bacteria have their own metabolism, drug studies can be confounded by the bacteria metabolizing the active compounds and indirectly altering the physiology of the worms (Cabreiro et al., 2013). To determine whether C22 directly alters *C. elegans* lifespan or if the observed effect is due to the bacteria metabolizing the drug, we tested the effect of C22 on worms given dead bacteria to eat (Beydoun et al., 2021). We find that in the absence of bacterial metabolism, C22 still extends N2 lifespan (Figure 4A) albeit to a smaller extent, and this lifespan extension is still dependent on *fmo-2* (Figure 4B), similar to what we observed with live bacteria (Figures 1D, 2D; Supplementary Table S1). We next looked at the effect of C22 on *fmo-2* induction using our *fmo-2*p::mCherry reporter worms fed live and dead OP50. We find that in the absence of bacterial metabolism, a lower concentration of C22 is sufficient to induce *fmo-2* (Figures 4C, D). This result may suggest that live bacteria metabolize C22 and the effective dose of C22 is much lower under live food. These results are consistent with the C22 compound itself having an *fmo-2-*inducing and lifespan extending mechanism, but one where lifespan is further extended when live bacteria are present.

## Conclusion

### C22 as a tool to induce sterility in *C. elegans*

From a screen of thousands of compounds tested for their effect on embryogenesis in worms, C22 was identified as a molecule that inhibits eggshell integrity (Weicksel et al., 2016). To determine the potential use of C22 as a FUdR alternative in *C. elegans* studies, we characterized its effect on various physiological measures. C22 is an effective inhibitor of embryogenesis under fed (Figure 1A) and DR (Figures 1B, C) conditions. However, exposure to C22 from egg or from young adulthood increases the lifespan of *C. elegans* fed live (Figures 1D, E) or dead (Figure 4A) bacteria. Our lifespan results are different from what was previously published (Weicksel et al., 2016) where lower concentrations of C22 (0.2 and 2.0 µM) were combined with FUdR to test the effects on lifespan. Since we were interested in using C22 as a FUdR alternative, we tested the lowest concentrations possible that were as effective at inducing sterility as well as FUdR, and thus focused on 3 and 5 µM of C22 without FUdR in this study. Our results illustrate the potential confounding effects of C22 on *C. elegans* lifespan independent of FUdR. Interestingly, unlike FUdR, where early exposure stunts development, C22 is most effective when worms are exposed to the compound before they begin producing eggs. The exposure of egg-laying adults to C22 before egg development is required for C22 to prevent egg-hatching.

### Limitations of the study

We show that C22 decreases pumping rate (Figure 2A) potentially resulting in a DR-like effect. However, we cannot conclude whether C22 directly results in a decrease in food intake or if the decrease in pumping is due to an indirect effect of C22 on the grinder function in the worms. We also do not know whether the bacteria are equally nutritious when exposed to C22 on the plates. To fully understand and quantify the effect of C22, future studies would be required to investigate quantitative food intake, defecation rate, food absorption and nutritional value of the bacteria exposed to C22. Also, since there are multiple methods to induce DR in *C. elegans,* one of the main limitations of the study is not testing for the interaction of C22 with other forms of dietary restriction (Lakowski and Hekimi, 1998; Kaeberlein et al., 2006; Lee et al., 2006; Bishop and Guarente, 2007; Panowski et al., 2007; Chen et al., 2009; Honjoh et al., 2009). It is well known that the type of bacteria that the worms consume can have different effects on various measures of health including fecundity and lifespan. Another limitation is focusing only on *E. coli* OP50 and not testing multiple types of bacteria relevant for the *C. elegans* community such as HT115 and HB101.

### Methods and caveats for differentiating test population from larva

Multiple methods have been utilized to limit progeny contamination in *C. elegans* studies, and each of them has caveats. Transferring worms daily (potentially over 10 days) increases the chances of physically “battering” the worms and confounding the results. Since worms undergo facultative vivipary (Chen and Caswell-Chen, 2004) when exposed to nutrient stress, daily transfers for DR studies without the use of a secondary method to inhibit internal hatching is not feasible since it will lead to a significant population loss. For this reason, DR studies in *C. elegans* generally use FUdR (Wang et al., 2019) to induce sterility and prevent vivipary (Chen and Caswell-Chen, 2004). However, the benefit of FUdR comes at a cost, as FUdR itself is implicated in longevity regulation (Cabreiro et al., 2013). Another method is the use of RNAi such as *pos-1* to induce embryonic arrest (Burton et al., 2011). A primary issue with this technique is a limited ability to use other RNAi to knockdown additional genes and study their function. Combining different RNAi, though technically possible, can lead to varying knockdown effects and in turn variable results. The last common tool used to prevent progeny contamination in *C. elegans* studies is the use of strains that have temperature dependent fertility/sterility (Spence et al., 1990; Beanan and Strome, 1992; Kodoyianni et al., 1992; Johnston et al., 2008). There are multiple issues with this method: 1) the confounding effect of temperature change on the physiology and lifespan of the worms (Miller et al., 2017; Vakkayil and Hoppe, 2022), 2) the effect of germline manipulation on lifespan (Hsin and Kenyon, 1999; Arantes-Oliveira et al., 2002) as seen with the extension of lifespan in the *glp-4* mutants, and 3) the requirement of crossing every mutant of interest into these specific strains.

### Interventions that impact fertility and their effect on lifespan

Many interventions that impact fertility in model organisms also alter lifespan (Ball et al., 1947; Merry and Holehan, 1979; Friedman and Johnson, 1988; Kenyon et al., 1993). In *C. elegans*, dimethyl sulfoxide (DMSO), a solvent used for polar and nonpolar pharmacological compounds in drug screens (such as C22), decreases brood size and extends lifespan (Frankowski et al., 2013). However, the concentration of DMSO (0.04%) used for the 5 µM C22 was an order of magnitude lower than the safe published concentration of 0.5% DMSO used to deliver compound in *C. elegans* research without impacting health and longevity (AlOkda and Van Raamsdonk, 2022). Interventions in the DR pathway have varying results on fertility and lifespan. Genetically, a loss-of-function mutation in the *daf-2* gene leads to a reduction in brood size and extension in lifespan (Hughes et al., 2007) while a gain-of-function in the FMO-2 protein, induced downstream of DR, extends lifespan without significantly impacting fecundity (Leiser et al., 2015). There are various forms of dietary restriction in *C. elegans* that effectively increase lifespan by modulating distinct and overlapping pathways (Kaeberlein et al., 2006; Lee et al., 2006; Bishop and Guarente, 2007; Panowski et al., 2007; Chen et al., 2009; Honjoh et al., 2009). In this work, we focus on the sDR (Greer et al., 2009) form of dietary restriction, since *fmo-2* is required for sDR-mediated lifespan extension (Leiser et al., 2015).

### C22, FMO-2, and DR

Knowing that C22 inhibits eggshell integrity (Weicksel et al., 2016), increases *C. elegans* lifespan, and decreases pumping rate (Figure 2A), plausibly resulting in a DR-like effect, we were interested in determining whether C22 interacts with the dietary restriction pathway. Our results show that C22 does not further decrease the pumping rate when combined with starvation (Figure 3C) and does not further increase sDR-mediated lifespan extension (Figure 3D), indicating that there is a potential interaction between C22 and at least one form of dietary restriction. Since *fmo-2* is induced downstream of DR, and it is necessary for sDR-mediated lifespan extension, we were interested in the potential interaction of C22 and *fmo-2*. Our results show that C22 induces the longevity enzyme FMO-2 (Figures 2B, C) and requires it for lifespan extension (Figure 2D) even in the absence of bacterial metabolism (Figure 4B). It is important to note that while *fmo-2* is required for 5 µM C22 to increase lifespan, exposure to overnight 5 µM C22 is not sufficient to induce *fmo-2.* Because a higher (100 µM) concentration of C22 induces *fmo-2* (Figure 2B) overnight, it is possible that a lower concentration (5 µM) of C22 for an extended period induces *fmo-2* and increases lifespan. Alternatively, it is possible that basal *fmo-2* expression, and not induction, may be required for C22 to extend lifespan. Future studies can determine the interaction of various concentrations of C22, exposure time and *fmo-2* induction in longevity regulation. Interestingly, the concentration of C22 required for mediating *fmo-2* induction is much lower when the bacteria are dead (Figures 4C, D), indicating that C22 as a compound has a direct effect on the worms partially independent of the bacteria metabolizing the drug. With regards to lifespan, it is possible that the effect of C22 is two-fold: a direct effect of the compound and an indirect effect of the metabolized compound. Since C22 has a greater effect on lifespan on live bacteria, it is plausible that bacterial metabolism of C22 or altered by C22 impacts *C. elegans* lifespan.

Overall, while C22 is unlikely to be a replacement for FUdR, its exact mechanism of longevity regulation and its potential interaction with other longevity pathways is worth further exploration.

## Data Availability

The original contributions presented in the study are included in the article/Supplementary Material, further inquiries can be directed to the corresponding author.
