# Supplementary material for: C22 disrupts embryogenesis and extends C. elegans lifespan
Source: Front Physiol. 2023 Sep 18;14:1241554. doi: 10.3389/fphys.2023.1241554 (PMC10544340; doi:10.3389/fphys.2023.1241554)
Supplement: Supplementary file 1 [file DataSheet1.docx]

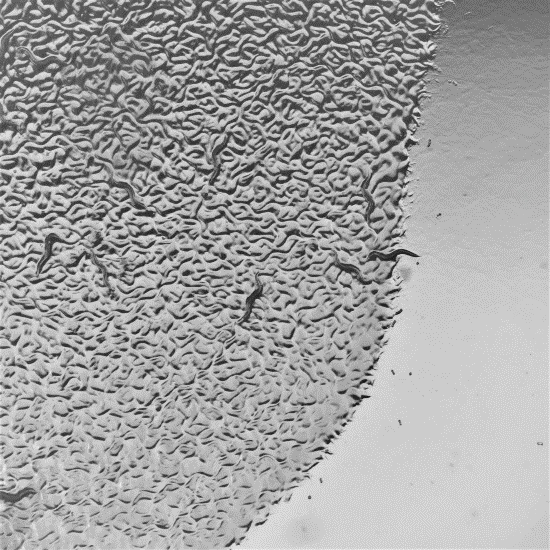

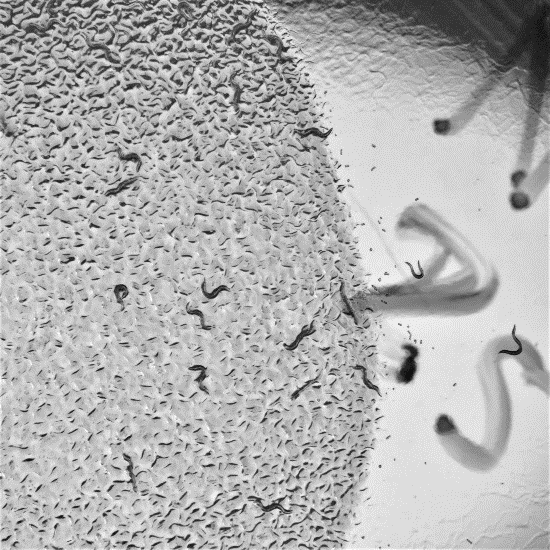


**B**

**A**

**Supplementary Figure 1. C22 disrupts embryogenesis at multiple temperatures.** Images of eggs not hatching from wildtype N2 worms grown on 5 μM C22 at **(A)** 15° and **(B)** 25° C.


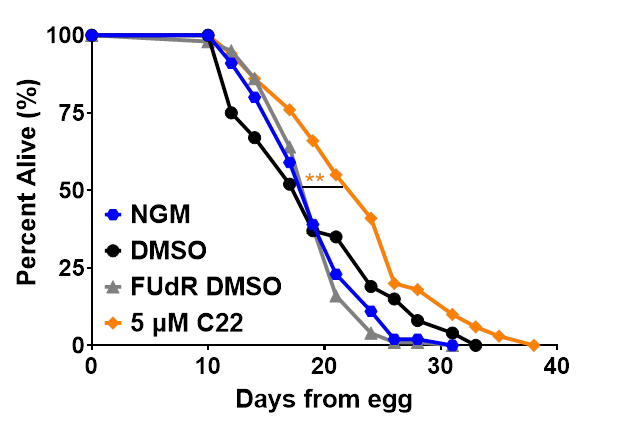


**Supplementary Figure 2. C22 increases wildtype N2 lifespan.** Lifespan of N2 worms on 5 µM C22 as compared to DMSO, NGM, and FUdR DMSO controls.


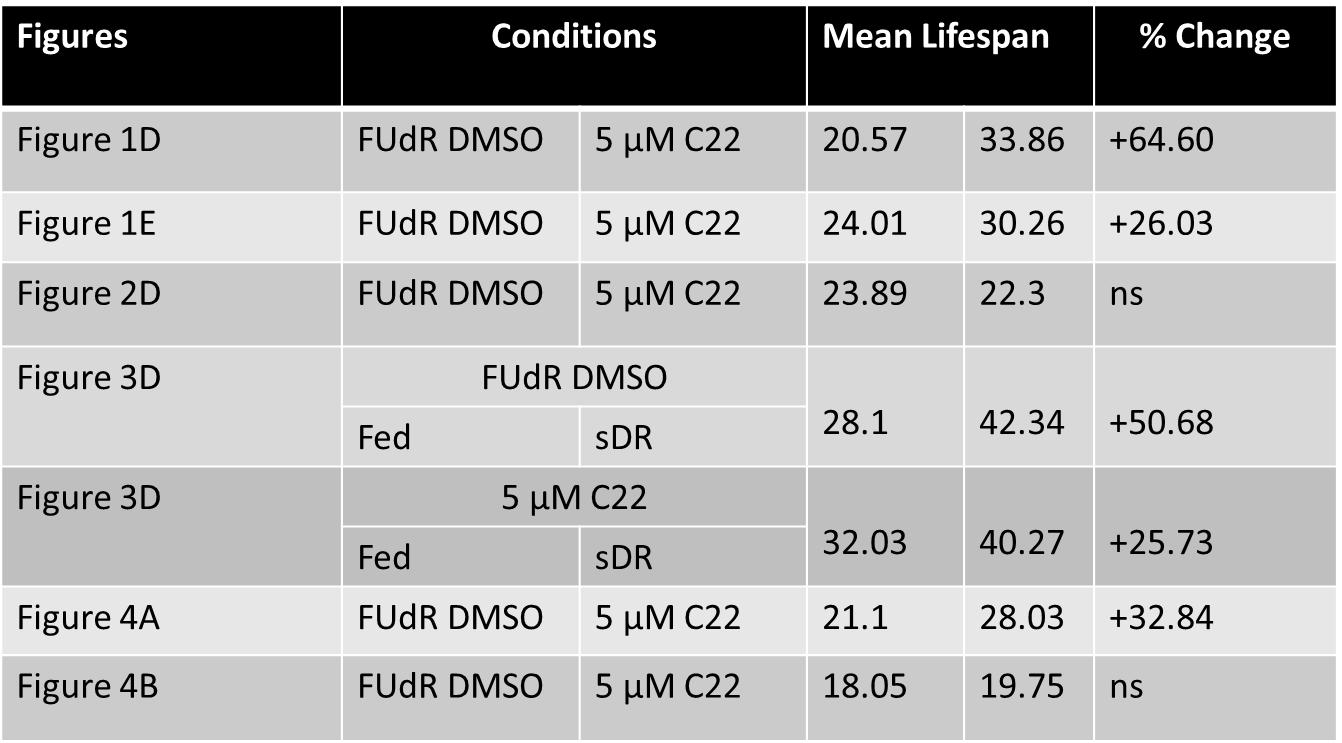


**Supplementary Table 1. Compilation of lifespan data showing percent change in lifespan across conditions.**
